# Synergistic effects of laccase and pectin on the color changes and functional properties of meat analogs containing beet red pigment

**DOI:** 10.1038/s41598-022-05091-4

**Published:** 2022-01-21

**Authors:** Kiyota Sakai, Yukihide Sato, Masamichi Okada, Shotaro Yamaguchi

**Affiliations:** Amano Enzyme Inc. Innovation Center, Kakamigahara, Japan

**Keywords:** Biotechnology, Enzymes, Proteins, Biochemistry, Carbohydrates, Polysaccharides

## Abstract

The widening gap between current supply of meat and its future demand has increased the need to produce plant-based meat analogs. Despite ongoing technical developments, one of the unresolved challenges of plant-based meat analogs is to safely and effectively imitate the appearance of raw and cooked animal-based meat, especially the color. This study aimed to develop a more effective and safe browning system for beet red (BR) in plant-based meat analog patties using laccase (LC) and sugar beet pectin (SBP). First, we investigated the synergistic effects of SBP and LC on BR decolorization of meat analog patties. We discovered that the red tones of LC-treated patties containing BR and SBP were remarkably browned after grilling, compared to patties that did not contain SBP. Notably, this color change by LC + SBP was similar to that of beef patties. Additionally, the hardness of LC-treated meat analog patties containing BR was higher than those that did not contain BR. Interestingly, the presence of SBP and LC enhanced the browning reaction and functional properties of meat analogs containing BR. This is the first report on a browning system for meat analogs containing BR using enzymatic methods to the best of our knowledge.

## Introduction

The widening gap between the current supply of meat and its future demand has increased the need to produce plant-based meat analogs as protein sources^1^. Developing better plant-based diets would address the current protein crisis and positively impact the environment and human health^1^. It has been estimated that progression from current omnivore diets to vegan and ovo-lacto vegetarian diets can achieve about 50% and 35% decrease of greenhouse gas emissions, respectively^2^. Also, numerous studies have reported the health benefits associated with the replacement of animal sources of protein with plant-based proteins, including reduced risks of type 2 diabetes, heart disease, and stroke^3,4^. Therefore, shifting the global food production system into a more sustainable one by developing plant-based analogs will not only address the future demand for protein sources but also support a healthy lifestyle and protect the environment.

Despite ongoing technical developments, the appearance, flavor, taste, and texture of plant-based meat analogs differ from those of traditional meat products. Among them, one of the unresolved challenges for plant-based meat analogs is their appearance, particularly their color^1,5^. Color is the first aspect of food products that consumers notice and a major contributor to consumer perception of taste and overall product acceptance^6^. Fresh, uncooked animal-based meats are bright red due to high oxymyoglobin content. When the meat is cooked, the metmyoglobin content increases, resulting in browning^7^. In contrast, most uncooked plant-based protein products are yellow or beige in color^1^. A major goal of research in this field is to simulate the typical browning of cooked animal-based meat by mimicking the red-to-brown color change in meat analogs^8^. However, few studies have investigated color changes in plant-based meat analogs^1,5,9^. Therefore, plant-based meat analogs are presently unable to meet the consumer standard established by animal-based meat in terms of appearance and color.

In recent studies on novel, plant-based meat analogs, the red color of the raw product has been obtained by the addition of (1) beet red (BR) pigment or (2) soy leghemoglobin^9^. (1) BR pigment mainly consists of betanin/betanidin extracted from *Beta vulgaris* ssp., and is traditionally used as a natural red pigment in the global food industry because of its safety and low cost^10–12^. *B. vulgaris* is one of the top ten most potent antioxidant-rich vegetables^13^. Betalains (including betanin/betanidin) with high antioxidant activity were suggested to have a protective function on certain oxidative stress-related disorders, such as cardiovascular diseases, cancer, aging, and neurodegenerative disorders^14^. Therefore, BR pigment is an attractive food coloring agent. However, BR as a coloring agent for meat analogs has two limitations. First, meat analogs containing BR might remain red after grilling as betanin is thermo- and photostable. Therefore, it is difficult for consumers and companies to judge their degree of doneness. Second, overheating meat analogs containing BR may result in an unacceptable yellow color^1,5,8,9^. (2) Leghemoglobin is chemically and structurally similar to myoglobin and imparts cooked-color characteristics similar to those of animal-based meat to meat analogs^9,15^. However, soy leghemoglobin is a genetically modified protein that is overexpressed in the methylotrophic yeast *Pichia pastoris*. Some consumers and companies have concerns regarding the risk of using genetically modified proteins in meat analogs.

Further research is needed to simulate the color changing of raw to cooked meat safely and more effectively. Presently, browning of meat analogs is achieved by adding coloring ingredients or other precursor substances. Specifically, caramel colors, malt extracts, reducing sugars, and amino acids are added, masking the red color and imparting the final product with a brown appearance^1,5^. However, consumers and manufacturers have safety concerns over using these additives. In addition, reducing sugars and amino acids may react to produce mutagenic and carcinogenic compounds, including acrylamide and heterocyclic amine, via the Maillard reaction^16,17^. Moreover, the additives fail to mask the red color of the pigment completely. Therefore, consumer and manufacturer perceptions have necessitated the development of a safe and more effective browning system for meat analogs.

The currently established dye and pigment decolorization methods are classified into four main categories: physical, oxidative, enzymatic, and biological methods^18^. Among them, the enzymatic method using laccase (LC; EC 1.10.3.2) has been applied in various industrial scenarios^19^. LCs are oxidases that couple the reduction of molecular oxygen to the one-electron oxidation of a wide variety of phenols^20–22^. The substrate-oxidizing activity of LCs can be enhanced using appropriate mediators. Consequently, LCs have been shown to catalyze the oxidation of a broad range of substrates such as phenol and its derivatives, benzenethiols, aromatic amines, and polycyclic aromatic hydrocarbons^23–25^. Therefore, they are widely used not only for the decolorization of synthetic dyes^26,27^ but also for bioremediation, detoxification, food processing, and biosensing^28^.

LC is used not only as a decolorizing enzyme but also as a crosslinking enzyme. Previous studies have reported that LC-catalyzed reactions formed pectin-chitosan films with hazardous gas removal abilities, arabinoxylan gels with higher gel abilities, and ferulate-modified pullulan with swelling properties^29–31^. In proteins, the exposed tyrosyl side chains serve as substrates for oxidation by LC, resulting in the spontaneous coupling of subsequent protein crosslinks (dityrosine)^19,32–35^. However, proteins are poor substrates for LC because of the intra-protein location of most tyrosine residues. Therefore, it has been reported that the presence of a macromolecular mediator enhances the LC-catalyzed protein crosslinking reaction^33^. These mediator-protein crosslinks enhanced the heat resistance, chemical bond strength, viscosity, gel strength, emulsifiability, and foamability of films or foods^33,34,36^. In our previous study on plant-based meat analog patties, the presence of sugar beet pectin (SBP) as a mediator also enhanced the protein-crosslinking activity of LC, thereby improving the physical and nutritional properties^37^.

In this study, we focused on developing a more effective and safe browning system for BR in plant-based meat analog patties using an enzymatic method. First, we investigated the synergistic effects of SBP and LC on BR decolorization of meat analog patties. Importantly, we discovered that the red tones of the LC-treated patties containing BR and SBP were remarkably browned after grilling, compared with patties that did not contain SBP. Additionally, the hardness of LC-treated meat analog patties containing BR was higher than those that did not contain BR. Interestingly, the presence of SBP and LC enhanced the browning reaction and functional properties of meat analogs containing BR. To the best of our knowledge, this is the first report on a browning system for meat analogs containing BR using enzymatic methods.

## Results and discussion

### Synergistic effect of LC and SBP on browning of meat analog patties containing BR

**Figure 1:**
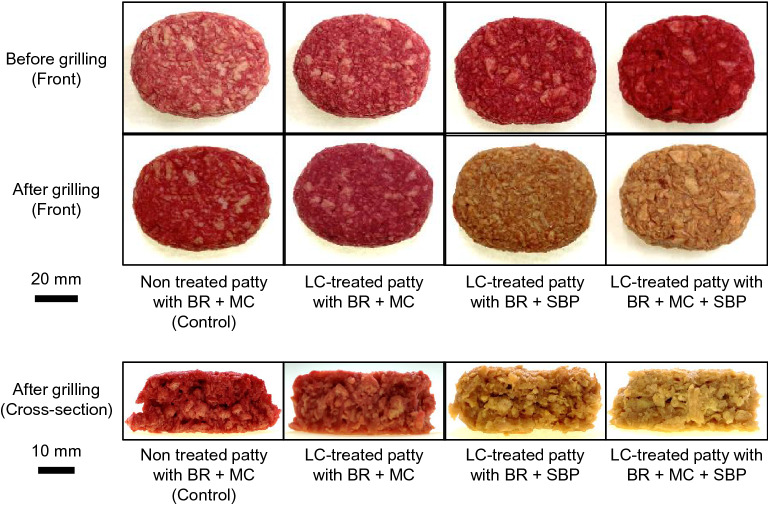
The appearance of the outside and inside of the meat analog patties before and after the grilling process.

To investigate whether SBP and LC have a synergistic effect on browning plant-based meat analogs, we added LC to pea-based patties containing BR and SBP (Fig. 1). Non-treated patties containing BR + methylcelullose (MC) were used as controls. After grilling, a red liquid resembling fresh meat juices exuded from the control patties, whereas the LC-treated patties containing BR + MC + SBP did not exude liquid (Fig. S1). The red color tones of non-treated and LC-treated patties with BR + MC were not affected by the grilling process (Fig. 1). The color of non-treated patties containing SBP did not change either (data not shown). In contrast, LC-treated patties containing BR + SBP or BR + SBP + MC developed a brown color during grilling.

**Figure 2:**
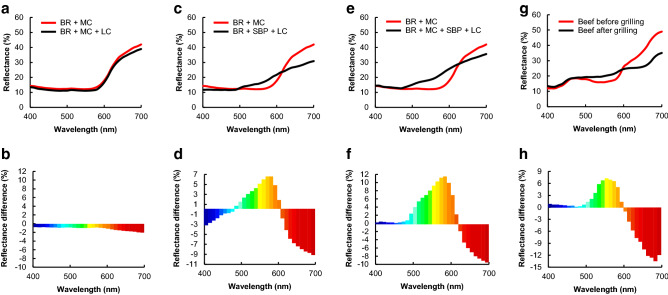
Reflectance of meat analog patties and beef patties. The reflectance values of (**a**,**b**) LC-treated patties containing BR + MC, (**c**,**d**) LC-treated patties containing BR + SBP, and (**e**,**f**) LC-treated patties containing BR + MC + SBP were measured using a colorimeter. Non-treated patties with BR + MC were used as controls. (**g**,**h**) Beef patties before and after grilling.

To investigate the color change in greater detail, the objective color attributes of the patties after grilling were measured using a colorimeter (Fig. 2). The color tone of the LC-treated patties containing BR + MC was slightly different from that of the control (Fig. 2a,b). Compared with the control, the reflectance of LC-treated patties containing BR + SBP or BR + SBP + MC increased in the 500–600 nm range and decreased in the 600–700 nm range (Fig. 2b,d). This indicates that the color of the patties changed from red to brown. Interestingly, the reflectance difference of LC-treated patties containing BR + SBP + MC was higher than that of LC-treated patties containing BR + MC (Fig. 2b,d). We also evaluated the color change of beef patties grilled under the same conditions. Similar to that of the LC-treated patties, the reflectance of beef patties also increased in the 500–600 nm range and decreased in the 600–700 nm range (Fig. 2g,h). The waveform comparisons of non-treated patties containing MC (control) with meat before grilling and LC-treated patties containing BR + SBP or BR + SBP + MC with meat after grilling were similar (Fig. S3). Next, the color of the meat analog patties was characterized using the L*a*b* coordinates (Table 1). It is indicated that a* (redness) decreased and b* (yellowness) increased in LC-treated patties containing BR + SBP or BR + SBP + MC after grilling. Similarly, a* decreased and b* increased under the same conditions in beef patties. The L* value (lightness) of LC-treated patties containing SBP was higher than that of patties without SBP. At the same time, the decrease rate in the L* value of animal-based patties was higher than plant-based patties. These findings of reflectance and L*a*b* values indicated that the browning system for BR by LC + SBP in plant-based patties could effectively imitate the color changes of animal-based patties.

**Table 1:** Objective color measurements (L*a*b*) coordinates of plant-based and animal-based patties.

Formulation	MC	SBP	BR	LC	Grilling process	L*	a*	b*
Non-treated patty with BR + MC (control)	+	−	+	−	Before	48.7 ± 1.7	48.5 ± 0.9	3.9 ± 1.1
					After	45.1 ± 2.1	47.9 ± 1.0	3.9 ± 0.5
LC-treated patty with BR + MC	+	−	+	+	Before	47.9 ± 3.5	48.4 ± 0.6	3.6 ± 1.0
					After	44.6 ± 1.7	47.0 ± 2.8	3.5 ± 0.5
LC-treated patty with BR + SBP	−	+	+	+	Before	53.7 ± 2.4	47.9 ± 2.4	3.8 ± 1.4
					After	52.7 ± 1.1	9.3 ± 1.1	43.5 ± 2.2
LC-treated patty with BR + MC + SBP	+	+	+	+	Before	55.6 ± 0.8	48.3 ± 3.2	3.7 ± 2.1
					After	54.1 ± 1.5	9.4 ± 1.6	46.6 ± 1.8
Beef patty	+	−	−	−	Before	54.8 ± 0.6	55.1 ± 1.7	3.5 ± 0.9
					After	45.2 ± 1.5	4.0 ± 1.3	39.7 ± 1.3

It is known that red betanin is oxidized by endogenous peroxidases in living red beets, resulting in color changes due to the formation of yellow betalamic acid and colorless cyclodopa^38–40^. Peroxidase is a hydrogen peroxide-requiring enzyme. Therefore, peroxidase decolorization of betanin has limited applicability in the food industry because hydrogen peroxide produces unacceptable off-flavors due to lipid oxidation, resulting in quality deterioration^41^. Peroxidases and LCs oxidize phenols using a similar oxidation mechanism, but only oxygen molecules are required for the laccase reaction^19^. The reflectance of LC-treated patties containing BR + SBP or BR + SBP + MC increased in the 500–600 nm (yellow) range and decreased in the 600–700 nm (red) range (Fig. 2d,f). These findings suggest that LC-catalyzed oxidation could degrade betanin (red) to betalamic acid (yellow) and cyclodopa (colorless), similar to the peroxidase-catalyzed reaction. The LC used in this study is a commercially available food-grade product that requires oxygen molecules alone to catalyze the reaction^19^. Betalamic acid has also been identified as a natural yellow pigment in plants^42^. Cyclodopa, a major metabolite derived from tyrosine, is a precursor of melanin in animals^43^ and betalain pigment in plants^44^. Humans have ingested foods containing these compounds for many years. Moreover, betalamic acid has been found beneficial in controlling diabetes mellitus by suppressing pancreatic amylase activities^45,46^. Therefore, these findings demonstrate a novel, effective, and safe browning process using LC and SBP for plant-based meat analogs containing BR pigment.

### Synergistic effect of LC and SBP on enhancing physical properties of patties

**Figure 3:**
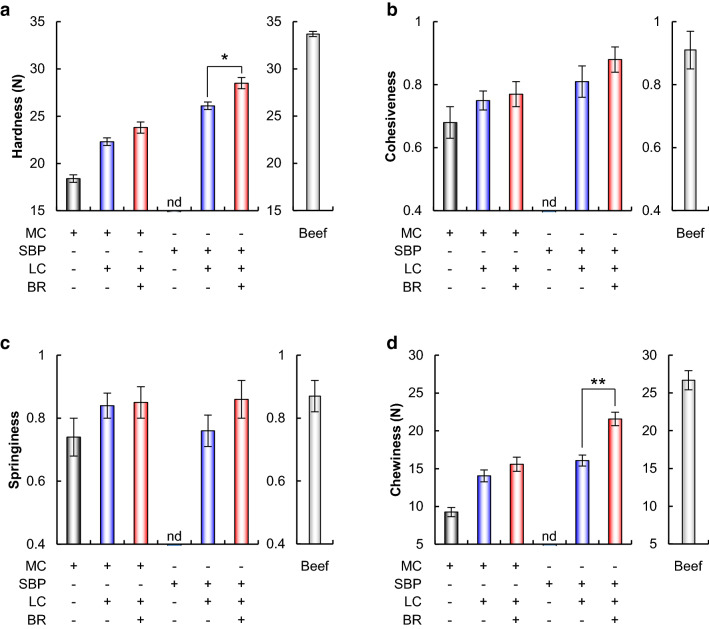
Texture profile analysis of meat analog patties containing BR and beef patties. (**a**) Hardness, (**b**) Cohesiveness, (**c**) springiness, (**d**) chewiness. Data are presented as mean ± standard deviation of five experiments. *p < 0.05, **p < 0.01, Student’s *t*-test.

The physical properties of meat analog patties containing BR were investigated (Fig. 3). Similar to a previous study^37^, non-treated patties containing SBP did not display moldability or binding ability. Interestingly, the hardness and chewiness values of LC-treated patties containing MC + BR were slightly higher than those of LC-treated patties containing MC (Fig. 3a,d). Moreover, the hardness and chewiness values of LC-treated patties containing SBP + BR were significantly higher than those of LC-treated patties containing SBP (Fig. 3a,d). While the hardness and chewiness values of these patties were lower than those of the beef patties, the cohesiveness and springiness values were similar to those beef patties (Fig. 3a,d). These findings indicated that LC enhanced the physical properties of plant-based patties containing BR, especially in the presence of SBP.

LC is also known as a protein-crosslinking enzyme. Generally, proteins are poor substrates for LC because of the intra-protein location of most tyrosine residues. It has been reported that the presence of a low-molecular phenolic mediator enhances the LC-catalyzed protein crosslinking reaction^47,48^. The mediator radicalized by LC can oxidize the intra-protein tyrosine residues where LC cannot directly attack, resulting in protein crosslinking^32^. Therefore, phenolic mediators such as vanillin, ferulic acid, and catechin could enhance the LC-catalyzed crosslinking reaction^49–51^. Interestingly, in this study, the presence of BR enhanced the hardness and chewiness attributes of the meat analog patties (Fig. 3). BR pigment consists mainly of betanin, which is a phenolic compound^10–12^. The presence of BR pigments could enhance LC-catalyzed crosslink reaction. Therefore, we investigated the effects of BR on crosslinks formed by LC, in detail. As shown in Fig. S2, the degree of LC-induced crosslinks increased in the following order: protein + SBP + BR > protein + SBP > protein. These findings suggest that BR could function as an LC mediator and enhance the degree of SBP-protein and protein–protein crosslinking induced by LC. Furthermore, LC in the presence of SBP caused a synergistic browning reaction in plant-based meat analog patties containing BR (Figs. 2 and 3). These findings suggest that SBP acts as a mediator of LC-induced browning in meat analogs containing BR.

Despite ongoing technical developments, the physical properties of existing plant-based meat analogs are still inferior to those of animal-based meat, especially in terms of texture and hardness^5,9^. It has been reported that the hardness values of plant-based patties are lower than that of animal-based patties^52^. In fact, the hardness values of LC-treated patties containing SBP + BR and LC-treated patties containing MC + BR was lower than that of beef patties grilled under the same conditions (Fig. 3a). Meat proteins generally exhibit a higher degree of shrinkage. The higher hardness values of animal-based patties have been suggested to be due to muscle protein denaturation, which leads to meat hardening^52,53^. The hardness and chewiness values of LC-treated patties containing SBP + BR and LC-treated patties containing SBP + MC + BR were similar to or higher than those of previously described plant-based meat analog patties^52,54–56^. This is because BR and SBP could function as mediators to enhance LC-induced protein crosslinking reactions (Figs. 3 and S2). These findings offer a novel strategy for improving the physical properties of plant-based meat analog patties and getting closer to the meat patty textures.

### Cooking loss of meat analog patties and beef patties

**Figure 4:**
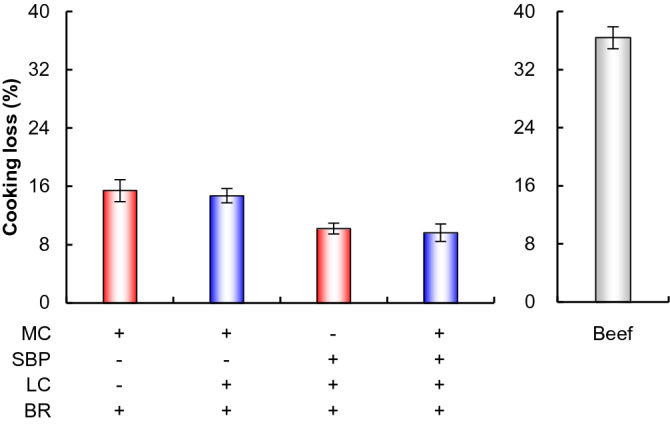
Cooking loss values of meat analog patties and beef patties. Cooking loss was calculated as the percentage weight difference between the dough before and after grilling. *p < 0.05, Student’s *t*-test.

We measured the cooking loss of the different formulations of plant-based meat analog patties (Fig. 4). Cooking loss, which reflects the degree of meat shrinkage during cooking, is an important indicator of the juiciness and yield of the final product. The cooking loss of patties containing SBP was lower than that of patties without SBP (Fig. 4). The presence of SBP in the formulation reduced the cooking loss by 4.4–5.2%, compared to that in the absence of SBP. Our previous study also reported a similar phenomenon in which LC-induced SBP-protein crosslinks reduced the cooking loss and improved the water/oil holding capacity of meat analog patties^37^. The use of MC as a binder is associated with the problem of low water/oil holding capacity^37,52,57^. In fact, a red liquid that looked like fresh meat juices exuded from non-treated patties containing BR + MC after the grilling process (Fig. S1). This is an unacceptable phenomenon for consumers and manufacturers. However, the LC-treated patties containing BR + MC + SBP exuded no such liquid (Fig. S1). This effect also occurred with LC-treated patties containing BR + SBP (data not shown). These findings indicate that enhancing the water holding capacity by adding SBP and LC to the formulation could overcome these unacceptable phenomena and enhance the juiciness of plant-based meat analog patties.

We also measured the cooking loss of beef patties (Fig. 4). The cooking loss value was 36.0%. This is in line with a previous study that reported a 40% cooking loss for commercial beef^58^. Moreover, Marchi et al. reported that cooking and volume loss values of animal-based burgers were higher than those of plant-based burgers^59^. This superiority of plant-based burgers could be explained by the greater dietary fiber content, as incorporating dietary fibers in meat products reduced their cooking loss^60^. Similarly, in this study, the cooking loss value of meat patties was significantly higher than that of plant-based meat analog patties containing dietary fibers such as MC or SBP (Fig. 4). Cooking loss value greatly affects food intake yield for consumers; therefore, plant-based meat analogs could be a major food source in the future. Moreover, dietary fiber in meat analogs is considered to play an essential role in preventing large bowel diseases, ischemic heart diseases, and diabetes mellitus^61^. Therefore, plant-based meat analog patties offer great promise in compensating the lack of the future protein source supply and contributing to disease prevention.

Furthermore, after the grilling process, the L* value of LC-treated patties with SBP + BR was higher than that of patties containing MC + BR (Table 1). Previous studies reported that L* values increased with increasing water/oil content of plant-based meat analog patties^62–64^. This is because small globules, such as water or oil, cause more light reflection^62,63^. Thus, it is suggested that LC-induced SBP-protein crosslinks decreased cooking loss, resulting in the increased L* value of LC-treated patties containing SBP. In contrast to the LC-treated patties with SBP + BR, the L* value of beef patties decreased after grilling (Table 1) as the cooking loss of beef patties was significantly higher than that of plant-based meat analog patties (Fig. 4). Furthermore, in this study, the pre-grilling L* value of LC-treated patties containing SBP + BR was higher than that of patties containing MC + BR (Table 1). This is because SBP itself has good amphiphilic properties, contributed by the protein moiety ferulate and acetyl groups, which impart hydrophobic properties, and the carbohydrate fraction, which imparts hydrophilic properties^65,66^. This could enhance the redness saturation of the patty before the grilling process. Color is the first and most crucial element that consumers notice in food products^6^. Therefore, the addition of SBP and LC could enhance the value of plant-based meat analog patties by enhancing their appearance before and after the grilling process.

### Putative enzymatic reactions that occurred in plant-based meat analog patties during grilling

Figure S4 shows the putative browning and SBP-protein crosslinking reactions involved in plant-based meat analog patties treated with LC. As shown in Reaction *1*, ferulates in SBP are oxidized by LC and O_2_, producing feruloyl radicals. This radical indirectly oxidized betanin by acting as an LC mediator (Reaction *2*). Also, betanin would be a substrate for LC because it is a typical phenolic compound (Reaction *3*). Betanin (red) is degraded to betalamic acid (yellow) and cyclodopa (colorless) via feruloyl radical-catalyzed indirect oxidation (Reaction *2*) and LC-catalyzed direct oxidation (Reaction *3*). Therefore, it is suggested that LC-catalyzed oxidation in the presence of SBP synergistically caused a red-to-brown color change in plant-based meat analogs containing BR. In the next step, cyclodopa is formed via Reactions *2* and *3*, which is further oxidized to form radicalized cyclodopa (Reaction *4*). This radical indirectly oxidizes the ferulates in SBP and the tyrosine residues in protein, producing feruloyl radicals and tyrosyl radicals (Reaction *5*). LC-catalyzed oxidation also produces these radicals (Reaction *6*). Finally, feruloyl radicals and tyrosyl radicals spontaneously crosslinked, subsequently producing SBP-protein crosslinks (Reaction *7*). Therefore, it is suggested that cyclodopa radical-catalyzed indirect oxidation (Reaction *5*) and the LC-catalyzed direct oxidation (Reaction *6*) act synergistically to form SBP-protein crosslinks, resulting in enhanced physical properties of plant-based meat analog patties. LC-catalyzed oxidation reactions in the presence of SBP effectively facilitated BR browning reactions and SBP-protein crosslinking reaction through the above-mentioned reactions.

## Conclusions

In this study, we developed an effective and safe browning system for BR in plant-based meat analog patties using LC + SBP. LC-treated patties containing BR and SBP showed remarkable browning after grilling and were comparable to beef patties. Additionally, LC-treatment improved the hardness of the patties containing BR. Thus SBP and LC enhanced browning as well as the functional properties of meat analogs containing BR. To the best of our knowledge, this is the first report on a browning system for meat analogs containing BR using enzymatic methods and would address the need for a more aesthetically appealing plant-based meat analog. Using our browning system, it would be possible to develop plant-based meat analogs that are more similar in appearance to meat patties and would also perform similarly to the meat patties during grilling. Such plant-based meat analogs would address the increasing demand for plant-based proteins.

## Materials and methods

### Materials

Granule-type pea-based textured vegetable protein (TVP) and sugar beet pectin (GENU pectin type BETA BI-J) were obtained from SANSHO Co., Ltd. (Tokyo, Japan). BR pigments and pea protein isolate (PPI) were purchased from KUMAMOTO BEET RED Co., Ltd. (Kumamoto, Japan) and Roquette Frères (Lestrem, France), respectively. Ground beef was obtained from a local supermarket in Nagoya (Japan). LC (LC-Y120; Amano Enzyme Inc., Nagoya, Japan) is a commercially available food-grade product. According to the manufacturer’s instructions, the optimal reaction temperature for LC is 60 °C, with a preferred temperature range of 40–70 °C (> 80% activity).

### Preparation of plant-based meat analogs

The base of the TVP matrix was prepared using TVPs and a binder (MC or SBP), followed by the addition of olive oil, BR, and PPI (Table S1). First, dried TVP was immersed in water (1:5 mass-to-volume ratio) for 2 h for hydration. After dehydrating the swollen TVP, it was mixed with 2.0% SBP or 2.0% MC at the final concentration. Thereafter, 5 g water, 8 g olive oil, 2.5 g PPI, and 0.5 g BR were added to each 25 g of TVP matrix. The samples were blended for 60 s using a hand blender. Thereafter, LC was added to the TVP matrix and blended for 60 s. The TVP matrix was molded (60 mm × 40 mm area and 25 mm height). The matrix was then cooked at 150 °C for 15 min and cooled to room temperature (20–25 °C) before being used for further analysis.

### Preparation of beef patties

Beef patties were prepared using ground beef, water, and MC. (Table S1). First, 2.0% MC at the final concentration and 5 g water were added to 35 g ground beef. The samples were blended for 60 s using a hand blender. Thereafter, the meat patties were molded. The matrix was then cooked at 150 °C for 15 min and cooled to room temperature (20–25 °C) before being used for further analysis.

### Color analysis

The color of the cooked meat analogs was measured using a colorimeter (Chroma Meter CM-700d/600d; Konica Minolta, Tokyo, Japan). The color analysis results were expressed according to the Commission International de l’Eclairage (CIE) system and reported as Hunter L* (lightness), a* (redness), and b* (yellowness).

### Cooking loss

The cooking method and conditions were determined based on the study by Pathare and Roskilly^67^. The patties were cooked at 150 °C for approximately 15 min, depending on the time taken for the temperature at the center of the meat analog to reach 80 °C. After cooking, the samples were cooled to room temperature (20–25 °C). Cooking loss was calculated as the percentage weight difference between the patty before cooking and after cooking, using the following formula: cooking loss (%) = [(W_1_ − W_2_)/W_1_] × 100; W_1_ is the weight of the meat analog before grilling (g), and W_2_ is the weight of the meat analog after grilling (g). The cooking loss of beef patties was estimated following a similar method.

### Texture profile analysis

Texture profile analysis was carried out using a COMPAC-100II (Sun Scientific Co., Ltd., Tokyo, Japan) equipped with a cylindrical probe of 31.4 mm area. After grilling, meat analogs and beef patties were prepared for the analysis and cut to a length of 15 mm in order to obtain homogeneous pieces of extrudates. The diameter of each sample was approximately 20 mm. A double compression cycle was performed at 1 mm/s until a recorded deformation of 50% was achieved. Five replicates were used for each sample. The following parameters were evaluated: hardness, the maximum force recorded during the first compression; cohesiveness, the area of work during the second compression divided by the area of work during the first compression; springiness, the distance recorded during the second compression divided by the distance of the first compression; and chewiness, hardness × cohesiveness × springiness.

### SDS-PAGE analysis of crosslinking

To investigate the synergistic effects of LC on the formation of crosslinks between proteins, betanidin, and SBP, the degree of crosslinking was measured by SDS-PAGE. 10% (v/v) pea + 1.0% RB pigment solution, 0.5% (v/v) SBP + 1.0% RB pigment solution, and 10% (v/v) pea + 0.5% (v/v) SBP + 1.0% RB pigment solution were assayed in 10.0-mL reaction mixtures containing 100 mM phosphate buffer (pH 7.0) and 250 units of LC. The reaction mixtures were incubated at 40 °C and then stopped by boiling at 100 °C for 5 min. Samples were prepared with an SDS-PAGE buffer (62.5 mM Tris–HCl pH6.8, 10% glycerol, 2% SDS, 5% DTT, and 0.002% BPB) under reducing conditions, and resolved on a 10–20% separating gel using an electrophoresis buffer (25 mM Tris, 19 mM glycine, and 0.1% SDS).

## Supplementary Information

Supplementary Information.
