# Supplementary material for: Synergistic effects of laccase and pectin on the color changes and functional properties of meat analogs containing beet red pigment
Source: Sci Rep. 2022 Jan 21;12:1168. doi: 10.1038/s41598-022-05091-4 (PMC8782913; doi:10.1038/s41598-022-05091-4)
Supplement: Supplementary file 1 — Supplementary Information. [file 41598_2022_5091_MOESM1_ESM.docx]

*Supplementary Files*

**Synergistic effects of laccase and pectin on the color changes and functional properties of meat analogs containing beet red pigment**

**Kiyota Sakai^*^, Yukihide Sato, Masamichi Okada, Shotaro Yamaguchi**

**
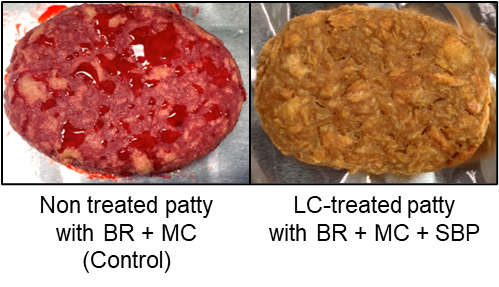
**

**Figure S1. The appearance of meat analog patties immediately after grilling.**

Non-treated patty with BR + MC (Control). LC-treated patty with BR +MC + SBP.


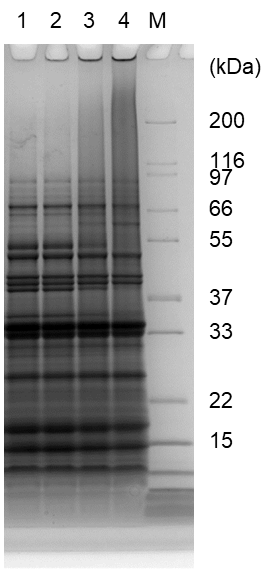


**Figure S2. Synergistic effect of LC combined with BR on the formation of protein crosslinks in meat analog patties.**

SDS-PAGE analysis was performed on pea protein and BR treated with 250 U of LC. Lane 1: non-treated pea protein; Lane 2: LC-treated pea protein; Lane 3: LC-treated pea protein and SBP; Lane 4: LC-treated pea protein, BR, and SBP.


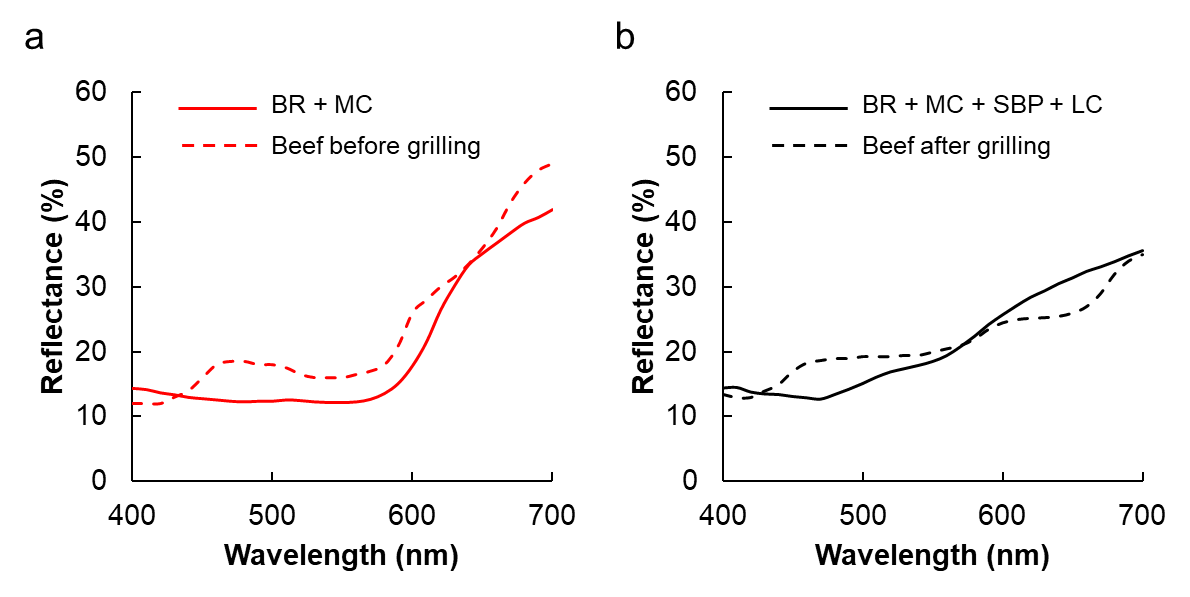


**Fig. S3. Reflectance of meat analog patties and beef patties.**

(a) The comparison between non-treated patty containing BR and beef patty before grilling. (b) The comparison between LC-treated patties containing BR + MC + SBP and beef patty before grilling.


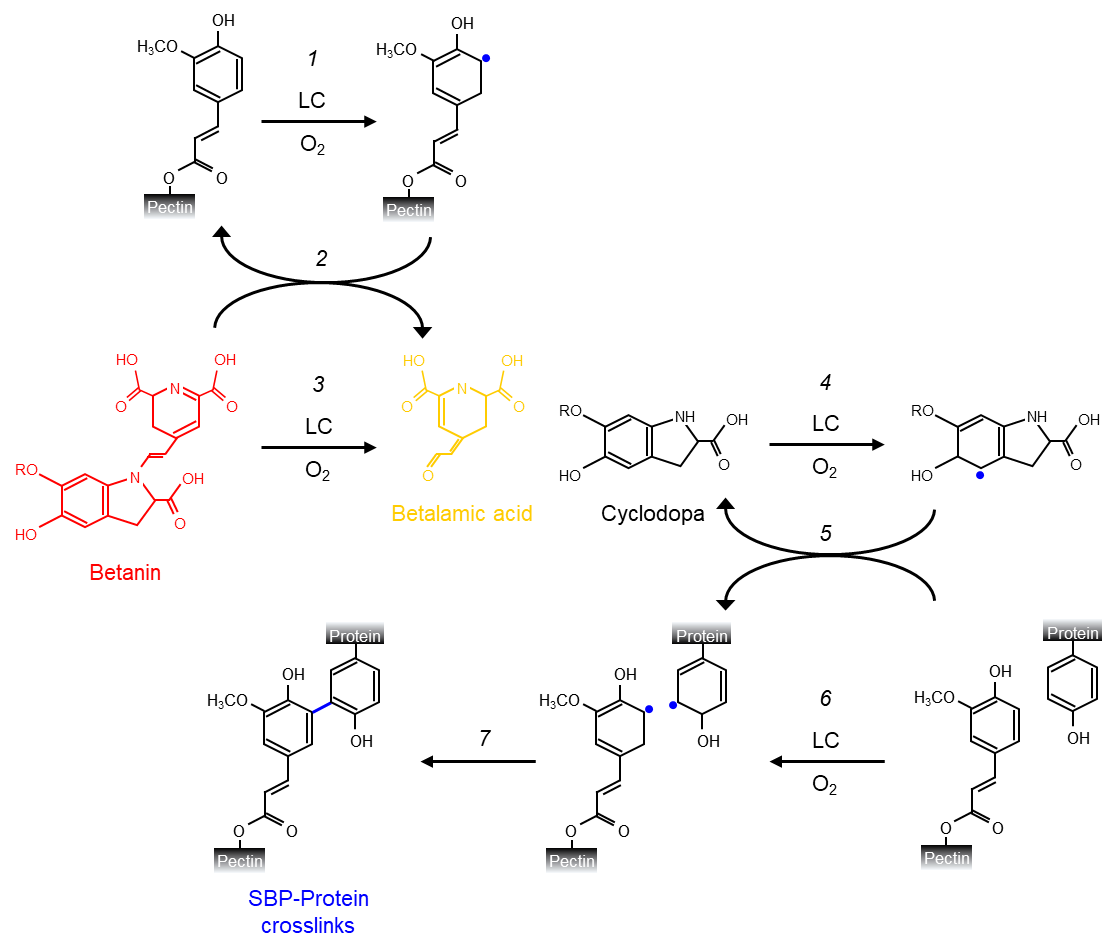


**Figure S4. Putative reactions involved in browning and crosslinking reactions in plant-based meat analog patties.**

Reaction *1*: Oxidation reaction catalyzed by LC; Reaction *2*: Indirect oxidation reaction induced by radicalized ferulic acids in SBP; Reaction *3*: BR browning reaction by LC; Reaction *4*: Oxidation reaction by LC; Reaction *5*: Indirect oxidation reaction by radicalized cyclodopa; Reaction *6*: oxidation reaction by LC; Reaction *7*: SBP-protein crosslinking reaction by phenoxy radicals.

**Table S1. Mixing amounts of additives or enzymes in plant-based meat analog patties.**

| Formulation | Wet TVP  (g) | Ground beef (g) | Water (g) | Oil (g) | PPI (g) | BR (g) | MC (%) | SBP (%) | LC (U/g TVP) |
| --- | --- | --- | --- | --- | --- | --- | --- | --- | --- |
| Non treated patty with BR + MC (Control) | 25 | - | 5 | 8 | 2.5 | 0.5 | 2 | - | - |
| LC-treated patty with BR + MC | 25 | - | 5 | 8 | 2.5 | 0.5 | 2 | - | 50 |
| LC-treated patty with BR + SBP | 25 | - | 5 | 8 | 2.5 | 0.5 | - | 2 | 50 |
| LC-treated patty with BR + MC + SBP | 25 | - | 5 | 8 | 2.5 | 0.5 | 2 | 2 | 50 |
| Beef patty | - | 35 | 5 | - | - | - | 2 | - | - |
